# Performance of the LIAISON^®^ SARS-CoV-2 Antigen Assay vs. SARS-CoV-2-RT-PCR

**DOI:** 10.3390/pathogens10060658

**Published:** 2021-05-26

**Authors:** Melanie Fiedler, Caroline Holtkamp, Ulf Dittmer, Olympia E. Anastasiou

**Affiliations:** Institute for Virology, University Hospital Essen, University of Duisburg-Essen, 45147 Essen, Germany; Melanie.Fiedler@uk-essen.de (M.F.); Caroline.Holtkamp@uk-essen.de (C.H.); Ulf.Dittmer@uk-essen.de (U.D.)

**Keywords:** SARS-CoV-2, COVID-19, antigen, LIAISON^®^ SARS-CoV-2 antigen assay, RT-PCR

## Abstract

We aimed to evaluate the LIAISON^®^ SARS-CoV-2 antigen assay (DiaSorin), comparing its performance to real-time polymerase chain reaction (RT-PCR) for the detection of SARS-CoV-2 RNA. 182 (110 PCR-positive and 72 PCR-negative) nasopharyngeal swab samples were taken for the detection of SARS-CoV-2. RT-PCR and antigen assay were performed using the same material. The sensitivity and specificity of the antigen assay were calculated for different cut-offs, with RT-PCR serving as the reference method. Stored clinical samples that were positive for other respiratory viruses were tested to evaluate cross-reactivity. One third (33/110, 30%) were falsely classified as negative, while no false positives were found using the 200 TCID_50_/mL cut-off for the SARS-CoV-2 antigen as proposed by the manufacturer. This corresponded to a sensitivity of 70% (60–78%) and a specificity of 100% (94–100%). Lowering the cut-off for positivity of the antigen assay to 22.79 or 57.68 TCID_50_/mL increased the sensitivity of the method, reaching a sensitivity of 92% (85–96%) vs. 79% (70–86%) and a specificity of 81% (69–89%) vs. 99% (91–100%), respectively. The antigen assay reliably detected samples with high SARS-CoV-2 viral loads (≥10^6^ copies SARS-CoV-2/mL), while it cannot differentiate between negative and low positive samples. Cross-reactivity toward other respiratory viruses was not detected.

## 1. Introduction

Since 2019, worldwide healthcare systems are being challenged by the COVID-19 pandemic, which is caused by the severe acute respiratory syndrome coronavirus (SARS-CoV-2). Because of the high infectiousness of the virus, it is important to test for it on a large scale, including with potential asymptomatic carriers [1]. Optimizing test strategies during the SARS-CoV-2 pandemic is an important and challenging goal. Hence, we aimed to evaluate the LIAISON^®^ SARS-CoV-2 antigen assay (Ag assay) (DiaSorin), comparing its performance to real-time polymerase chain reaction (RT-PCR) for the detection of SARS-CoV-2 RNA in nasopharyngeal swabs.

The LIAISON^®^ SARS-CoV-2 Ag assay is a chemiluminescence sandwich-immunoassay (CLIA)-based technology for the determination of nucleocapsid antigen from SARS-CoV-2 samples in upper respiratory specimens. We used two different RT-PCRs: The RealStar^®^ SARS-CoV-2 RT-PCR kit (Altona), which targets the SARS-CoV-2 genes S (spike) and E (envelope), and the Alinity m SARS-CoV-2 Assay (Abbott), which targets the SARS-CoV-2 genes RdRp (RNA dependent RNA polymerase) and N (nucleocapsid).

RT-PCR is actually considered the gold standard for the detection of SARS-CoV-2 [2]. Nevertheless, there are some disadvantages with using RT-PCR. It is time-consuming, expensive, prone to contamination, and trained laboratory staff as well as specialized equipment are needed [1]. In addition, during the pandemic, reagents have often been scarce or even unavailable [3], and the demands on specialized laboratories (providing RT-PCR diagnostics) consequently increased. Hence, we evaluated whether using an antigen assay could be an adequate back up system for the RT-PCR. A benefit of using the antigen assay is the availability of the LIAISON^®^ in many laboratories, which could potentially expand our diagnostic capabilities. Previous studies have already compared different antigen rapid diagnostic tests with RT-PCR: Corman et al. [3] showed that antigen rapid diagnostic tests were able to detect SARS-CoV-2 viral load of between 10^6^ and 10^7^ copies per swab.

The objective of our study was the comprehensive evaluation of the LIAISON^®^ SARS-CoV-2 antigen assay for the detection of SARS-CoV-2 in respiratory tract samples, focusing on a head-to-head comparison with the gold-standard method, RT-PCR.

## 2. Results

### 2.1. The SARS-CoV-2 Nucleocapsid Antigen Concentration Remains Stable over 3 Days

**Figure 1:**
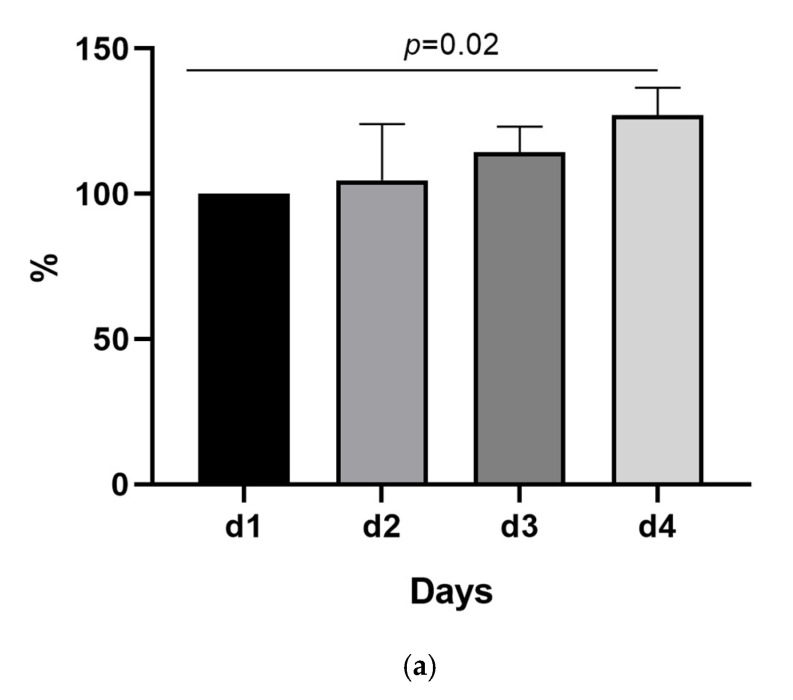

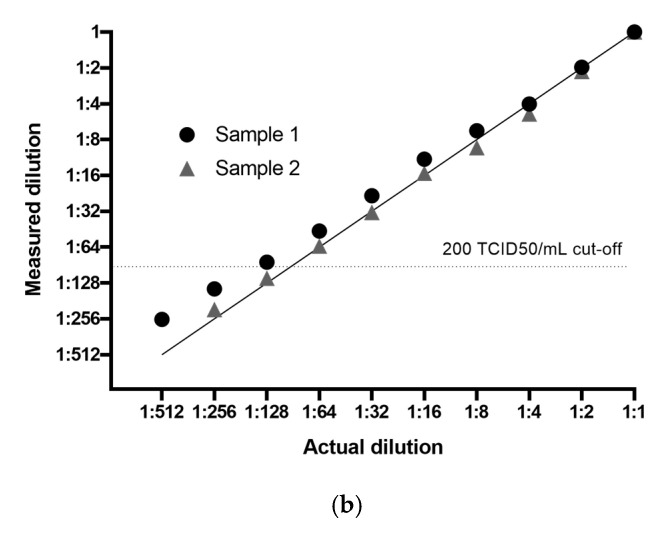
(**a**) SARS-CoV-2 antigen concentration normalized to baseline values over a four-day period. Values are presented as median (interquartile range). The comparison between the groups was performed using the Friedmann test (*p* = 0.02). A significant difference can be observed when comparing the first to the fourth day (*p* = 0.02). All other comparisons did not reach statistical significance. (**b**) Serial dilution and linearity of the assay. The line represents the actual dilution values on the *x*- and *y*-axis, and the triangles and dots represent the measured values for each dilution. The triangles and dots present the average value from two technical duplicates. The assay demonstrates linearity, especially for values over the 200 Tissue Culture Infectious Dose (TCID_50_/mL) cut-off.

According to the manufacturer’s instruction for the LIAISON^®^ SARS-CoV-2 antigen assay, the deactivation of the sample should be performed within 12 h after sampling. Collecting, distributing, and processing the samples within this timeframe can be a logistical challenge for many healthcare providers. To test if the antigen measurement remains stable over a longer timeframe, we used samples from 10 patients, with 400 µL of each sample being inactivated from days 1 to 4. The original material was kept at 4 °C for the four-day period. All samples had a viral load of >10^6^ copies/mL, ranging from 1.5 × 10^6^ to 1.9 × 10^8^ copies/mL. As shown in Figure 1a, the antigen levels remained remarkably stable over the first three days, which is in line with our daily routine in the laboratory setting. When comparing the first and fourth day, we observed a significant increase in the normalized Tissue Culture Infectious Dose (TCID_50_/mL) values of 27%, with an interquartile range of 0.5–37% (*p* = 0.02). All other comparisons between different time-points did not reach statistical significance.

To test the linearity of the assay, we performed a series of dilutions (1:2, 1:4, 1:8, 1:16, 1:32, 1:64, 1:128, 1:256 and 1:512) for two samples. Each dilution and the undiluted sample were tested in duplicate. The assay demonstrated linearity, especially for values over the 200 TCID_50_/mL cut-off (Figure 1b).

### 2.2. A Third of the Samples Was Falsely Classified as Negative Using the 200 TCID_50_/mL as Cut-Off

We tested 182 nasopharyngeal swab samples by PCR and the LIAISON^®^ SARS-CoV-2 antigen assay to detect SARS-CoV-2. The PCR was considered the gold standard for the classification of the antigen results as true/false negative and positive. Our cohort consisted of 110 SARS-CoV-2 PCR-positive and 72 negative samples. A third (33/110, 30%) of the samples were falsely classified as negative with the antigen assay. No false positives were found using the cut-off of 200 TCID_50_/mL for the SARS-CoV-2 antigen as shown in Table 1, corresponding to a sensitivity of 70% (60–78%), a specificity of 100% (94–100%), a positive predictive value (PPV) of 1 (0.94–1), and a negative predictive value (NPV) of 0.69 (0.59–0.77).

### 2.3. Lowering the Cut-Off for the Antigen Assay Can Significantly Increase the Sensitivity of the Method

**Figure 2:**
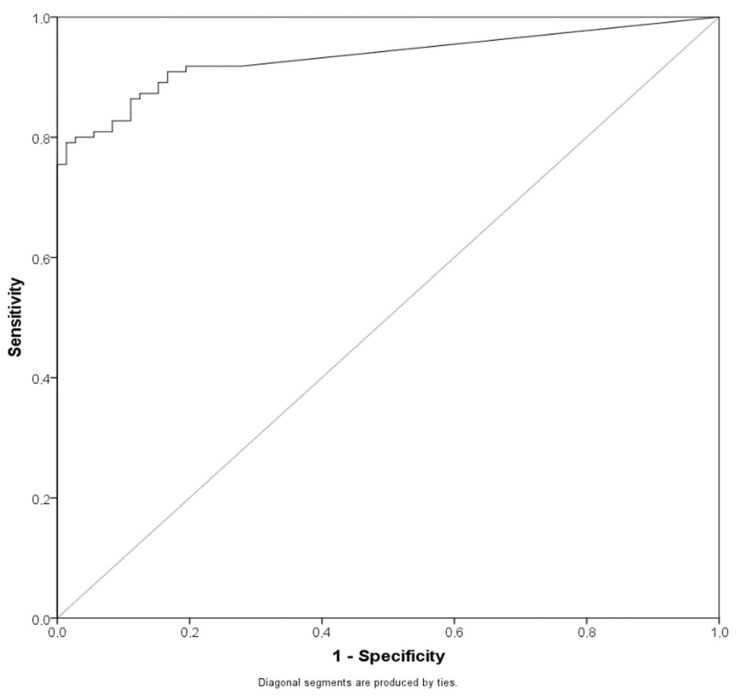
Receiver operating characteristic (ROC) curves for SARS-CoV-2 antigen values of SARS-CoV-2 RT-PCR-positive samples.

We investigated whether using a different cut-off for sample positivity could improve sensitivity. The 100 TCID_50_/mL was selected for evaluation because the range from 100–200 TCID_50_/mL is defined as borderline in newer versions of the information sheet for the LIAISON^®^ SARS-CoV-2 antigen assay. In addition, a receiver operating curve (ROC) was calculated to pinpoint optimal cut-offs (Figure 2). The area under the curve (AUC) was 0.932 (0.895–0.969) (*p* < 0.001).

Sensitivity, specificity, PPV, and NPV were calculated for these four different cut-offs (Table 2). Raw values for Table 2 can be found in the Supplementary Section. The first two cut-offs were selected because they provided a sensitivity of over 90%. The use of 22.79 or 23.56 TCID_50_/mL increased the sensitivity, increasing the correct classification of positive samples from 77/110 (70%) to 101 (92%) and 100/110 (91%), respectively. It also led to the misclassification of 14/72 (19%) and 12/72 (17%) negative samples as false positive, respectively. The third cut-off (57.68 TCID_50_/mL) was calculated according to the maximum value of the Youden Index for providing the best combination of sensitivity (79%) and specificity (99%) [4]. The fourth (100 TCID_50_/mL) provided a specificity of 100%, while the sensitivity was only 75%, leading to a misclassification as false negative for 27 of the 110 positive samples.

### 2.4. The LIAISON^®^ SARS-CoV-2 Antigen Assay Can Reliably Detect Samples with High SARS-CoV-2 Viral Load, While the Test Cannot Reliably Differentiate between Negative and Low Positive Samples

**Figure 3:**
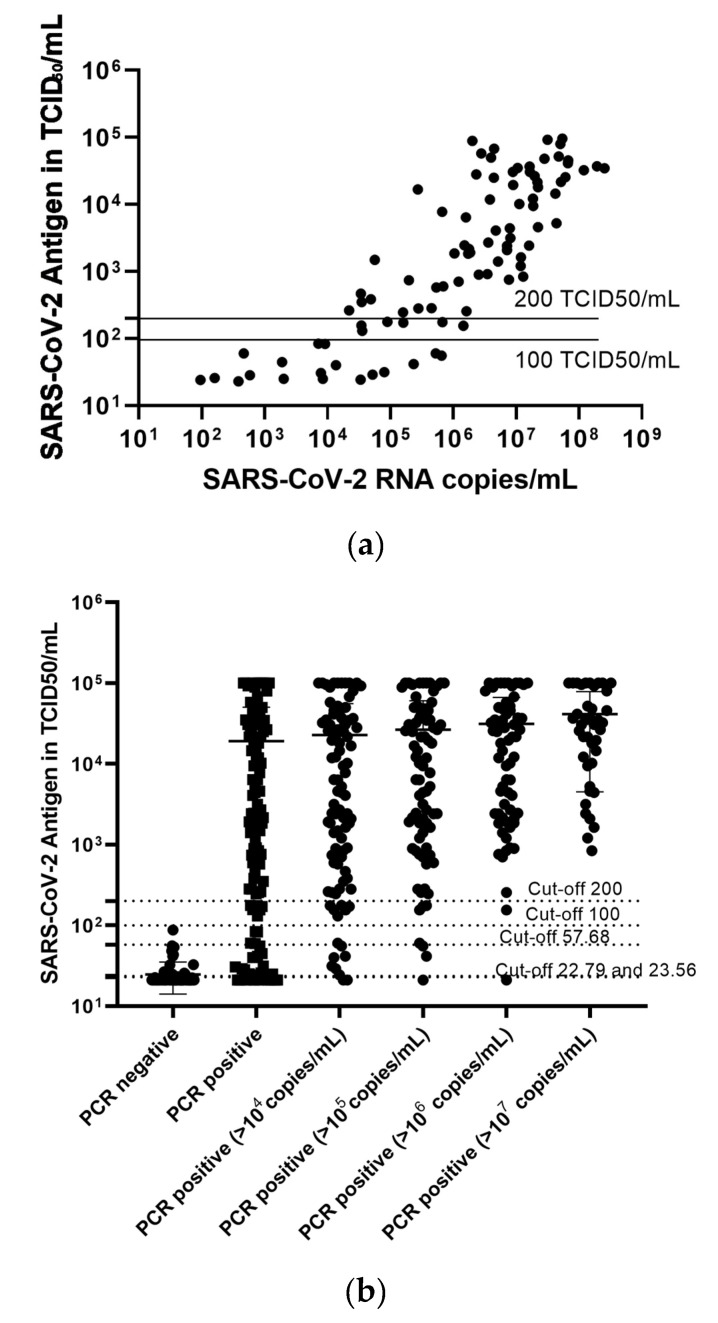
A linear relationship was observed between the log values of antigen concentration and viral load (*n* = 93, values outside the quantified range of antigen concentration (22–100,000) were excluded) (**a**). Stratifying the samples according to their PCR status as negative (*n* = 72), positive (*n* = 110), as well as the more differentiated view of the positive samples, including low positive samples from >10^4^ copies/mL (*n* = 92), moderately high positive samples from >10^5^ copies/mL (*n* = 79), high positive samples from >10^6^ copies/mL (*n* = 66), and very high positive samples from >10^7^ copies/mL (*n* = 43), revealed that the antigen assay can differentiate between negative samples and samples with high viral load, while a significant overlap can be observed between negative and low positive samples (**b**).

Next, we looked at a potential correlation between the antigen levels in TCID_50_/mL and the viral load measured through SARS-CoV-2 PCR. A linear relationship was observed between the logarithms of antigen concentration and viral load as shown in Figure 3a. After excluding values outside the quantified range of antigen concentration (22–100,000), we calculated a linear regression model (r^2^ = 0.836, *p* < 0.001) with the following equation: log(viral load) = 2.51 + [1.1 × log(antigen)].

Plotting the antigen levels in PCR-negative and all PCR-positive samples, as well as the more differentiated view of the positive samples with more than 10^4^ (low positive), 10^5^ (moderately high positive), 10^6^ (high positive), and 10^7^ (very high positive) copies of SARS-CoV-2 per mL revealed that the antigen assay can reliably differentiate between negative samples and samples with high viral loads. However, we observed a significant overlap between negative and low positive samples (Figure 3b).

### 2.5. Testing Other Materials by LIAISON^®^ SARS-CoV-2 Antigen Assay: Possible but Suboptimal

One of the advantages of RT-PCR is the detection of viral RNA in different materials. We aimed to test whether the LIAISON^®^ SARS-CoV-2 antigen assay could also be used to test different respiratory tract materials. We evaluated 28 pharyngeal wash samples, 8 bronchoalveolar lavage (BAL) samples, and 1 sputum sample (data not shown). The measurement could not be completed due to pipetting errors in two BAL samples and the sputum sample. We included 24 RT-PCR-positive pharyngeal wash samples, with a viral load ranging from 78 to 1.7 × 10^5^ copies/mL and 4 negative ones. Using the 200 TCID_50_/mL cut-off for negativity, all 4 PCR-negative samples were correctly classified as negative, while 23 samples were misclassified as negative and 1 sample with a viral load of 1.7 × 10^5^ copies/mL was correctly classified as positive. Using the 57.68 TCID_50_/mL cut-off for negativity all samples, positive and negative, were classified as positive. From the remaining 6 BAL samples, 1 was positive with a viral load of 6 × 10^4^ copies/mL and 5 negative. When using the 200 TCID_50_/mL cut-off for negativity, all BAL samples were negative, while when using the 57.68 TCID_50_/mL cut-off, all samples were classified correctly.

Samples positive for endemic human coronaviruses (HCoVs) (HCoV NL63, HCoV OC43, HCoV 229E, HCoV HKU), influenza A and B, respiratory syncytial virus (RSV), and samples from patients with detectable Epstein–Barr Virus (EBV) in blood were tested using the LIAISON SARS-CoV-2 antigen assay (Ag assay) (data not shown). We observed no cross-reactivity, taking into account the manufacturer´s cut-off of 200 TCID_50_/mL. The RSV, influenza, HCoV NL63, HCoV OC43 had a value of less than 22 TCID_50_/mL, while the TCID_50_/mL values for HCoV 229E and HKU amounted to 22.2 and 49.2 TCID_50_/mL. The TCID_50_/mL values of the samples of EBV positive patients ranged from 30.9 to 35.7 TCID_50_/mL.

Lastly, we tested 10 nasopharyngeal swab samples (viral load >10^6^ copies/mL) from patients carrying the B.1.1.7 variant. All had the detectable SARS-CoV-2 antigen when taking the 200 TCID_50_/mL cut-off into account (data not shown).

## 3. Discussion

In this study, we compared the performance of the LIAISON^®^ SARS-CoV-2 antigen assay vs. the gold standard for SARS-CoV-2 detection, RT-PCR [2]. Our cohort consisted of individuals tested by the Public Health Department of Essen. In case of outbreaks, this also included asymptomatic individuals. Unfortunately, we cannot provide precise information since none is available to us. The absence of such information, however, is not unusual for samples sent to a laboratory for SARS-CoV-2 testing, but it is still a limitation of the study.

A third of the samples with detectable SARS-CoV-2 RNA by RT-PCR were falsely classified as negative, with the antigen test taking the manufacturer’s recommended cut-off of 200 TCID_50_/mL. The antigen test is, indeed, inferior to the RT-PCR in terms of sensitivity, which is not surprising. RT-PCR is considered the gold standard for SARS-CoV-2 detection. However, even the gold standard is not without disadvantages: it is expensive, time consuming, requires trained laboratory staff as well as specialized equipment [1], and may not always be available (for example, due to lack of reagents) [3].

However, no false positives were found, amounting to 100% specificity and 70% sensitivity. Antigen tests, especially antigen rapid diagnostic tests, are known to be less sensitive than RT-PCR [5] and may be comparable or inferior in terms of sensitivity to other rapid detection methods, such as loop-mediated isothermal amplification assay [6,7]. The WHO recommends the use of antigen tests with sensitivity and specificity greater than 80% and 97%, respectively [8]. Using a ROC analysis, we identified different cut-offs, optimized for maximal sensitivity and/or a combination of sensitivity and specificity, and compared three of them. As expected, lowering the cut-off for the antigen assay increased sensitivity. In fact, the two lowest cut-offs increased sensitivity to over 90%. This is significantly higher than the sensitivity of various antigen rapid diagnostic tests, which range from 73% to 82%, as reported in a meta-analysis by Brümmer, et al. [5], and may be as low as 62.5% in the case of asymptomatic subjects. However, the higher sensitivity, attained by lowering the cut-off, comes at the cost of decreased specificity. Using the cut-off between 22 and 23 TCID_50_/mL was found to lead to false positive results for almost a fifth of the negative samples. The third and optimal cut-off (57.68 TCID_50_/mL), calculated according to the maximal Youden Index, showed an acceptable combination of sensitivity (79%) and specificity (99%). This cut-off may be considered by the manufacturer. In addition, this cut-off roughly fulfilled the WHO standard for SARS-CoV-2 antigen tests [8]. Recently, a Japanese group evaluated the performance of another quantitative, fully automated antigen assay with nasopharyngeal swabs. The Lumipulse^®^ SARS-CoV-2 Ag test (Fujirebio Inc., Tokyo, Japan) is also a chemiluminescence enzyme immunoassay, which tests the nucleocapsid antigen [9]. They described a sensitivity of 91.7% and a specificity of 98.5% using an optimized cut-off. The specificity is roughly in line with our results, but the sensitivity is substantially higher. This may reflect the superiority of the Fujirebio assay compared to the LIAISON^®^ SARS-CoV-2 antigen assay. A head-to-head comparison of the two assays would be necessary to conclusively evaluate the point, since the two studies focused on different cohorts.

Virus concentrations of 10^6^ and 10^7^ copies/mL are almost always detected using the cut-off of 57.68 TCID_50_/mL for the LIAISON^®^ SARS-CoV-2 antigen assay. Virus isolation is possible in only 20% of samples with virus concentrations within this range [10]. In addition, Corman et al. [3] suggested that a negative antigen test result obtained a week after the first symptoms is associated with loss of infectiousness. The LIAISON^®^ SARS-CoV-2 antigen assay could probably be useful on this aspect and could be the basis of lifting isolation in long-term hospitalized COVID-19 patients. However, more studies are necessary to correlate TCID_50_ and viral loads with absence of infectiousness in vitro. Hence, testing in cell culture might be an interesting additional investigation.

According to the manufacturer´s instructions for the LIAISON^®^ SARS-CoV-2 antigen assay, it is necessary to perform the sample deactivation within 12 h after taking the sample. Practical knowledge showed that this is usually impossible in a clinical daily routine. To overcome this problem, we evaluated antigen stability over time and found that the antigen measurement remains stable over three days. For other antigens, stability over time has been shown before, e.g., the stability of the hepatitis B surface antigen over 12 months stored at −20 °C [11].

Apart from nasopharyngeal swab samples, which are most frequently used in the daily routine [12], SARS-CoV-2 diagnostics includes the evaluation of other materials. Here, the LIAISON^®^ SARS-CoV-2 antigen assay performed suboptimally. It could not reliably detect SARS-CoV-2 in other materials, such as bronchoalveolar lavage, pharyngeal wash, or sputum, which is a limitation of this test in comparison to RT-PCR. Cross-reactivity toward other respiratory viruses was not observed. This underlines the reliability of the LIAISON^®^ SARS-CoV-2 antigen assay, since at the beginning of the pandemic, false positive results were generated by RT-PCR due to cross-reactivity [13].

In conclusion, the LIAISON^®^ SARS-CoV-2 antigen assay can reliably detect samples with high SARS-CoV-2 loads. Hence, the antigen assay might be a useful back-up system if performing RT-PCR is not an option (for example, due to lack of reagents). In addition, it might be a helpful tool for lifting the isolation of hospitalized patients.

## 4. Materials and Methods

We included 182 nasopharyngeal swabs for the detection of SARS-CoV-2, tested from 16 November to 21 December 2020 at the Institute for Virology, University Hospital Essen, in our study. Our cohort consisted of individuals tested by the Public Health Department of Essen. The sample collection was performed when patients informed the Public Health Department of their COVID-19-specific symptoms. Generally, this was briefly after the onset of symptoms. In case of outbreaks, this also included asymptomatic individuals.

The swab collection kits contained a viral transport medium, which was used for both the PCR and the detection of SARS-CoV-2 antigen, as described below.

Detection of SARS-CoV-2 RNA was performed using the RealStar^®^ SARS-CoV-2 RT-PCR kit (Altona Diagnostics, Hamburg, Germany), which targets the SARS-CoV-2 genes S (spike) and E (envelope) for 155 samples and Alinity m SARS-CoV-2 Assay (Abbott, Wiesbaden, Germany), which targets the SARS-CoV-2 genes RdRp (RNA dependent RNA polymerase) and N (nucleocapsid) for 27 samples. For the Altona assay, the Ct values corresponding to the E-gene were taken into account for further analysis. Furthermore, we tested two SARS-CoV-2 standards provided by INSTAND (Berlin, Germany) of 10^6^ and 10^7^ SARS-CoV-2 copies/mL, which corresponded to Ct values of 23.11 (E-gene, Altona) and 22.42 (Alinity) and 20.18 (E-gene, Altona) and 18.67 (Alinity), respectively. We diluted the 10^6^ standard and measured the Ct values corresponding to 10^5^ and 10^4^ copies/mL, amounting to Ct 26.3 and 31.4 (E-gene, Altona) and 25.1 and 28.74 (Alinity), respectively. Linear regression was performed for both series of values, and the viral load in copies/mL was calculated based on the equation deriving from it.

SARS-CoV-2 antigen detection was performed using the LIAISON^®^ SARS-CoV-2 Ag assay (Diasorin, Saluggia, Italy), a chemiluminescence sandwich-immunoassay (CLIA)-based technology for the determination of nucleocapsid antigen from SARS-CoV-2 samples in upper respiratory specimens. Samples positive for endemic coronaviruses (HCoV NL63, HCoV OC43, HCoV 229E, HCoV HKU), influenza A and B, RSV, and samples from patients with detectable EBV in blood were tested using the LIAISON SARS-CoV-2 Ag assay to evaluate cross-reactivity. The cut-off for sample positivity given by the manufacturer is 200 TCID_50_/mL. The assay was performed according to the manufacturer´s instructions with two notable exceptions. For the inactivation of the samples, we mixed a 400 µL sample with a 400 µL inactivation buffer, instead of using 1 mL plus 1 mL. Many swab sample collection kits do not contain enough viral transport media to allow for the testing of the sample with the antigen assay, RT-PCR, and a potential retesting (for example, in case of inhibition or pipetting errors). The applied volume was within the range of the minimum sample volume given by the manufacturer (100 µL sample + 300 µL dead volume). Furthermore, we used samples taken up to 36 h before inactivation, since this reflects laboratory and clinical routine more accurately. The samples were cooled during transport and storage. The ethics committee of the medical faculty of the University of Duisburg-Essen approved the assessment of test samples for the improvement of diagnostic procedures (20-9512-BO). Statistical analysis was performed using SPSS software (v23, SPSS Inc., Chicago, IL, USA), GraphPad Prism 6.0 (GraphPad, CA, USA) and the platform VassarStats (http://vassarstats.net (accessed on 23 March 2021). Two-tailed *p* values less than 0.05 were considered to be statistically significant.

## Figures and Tables

**Table 1:** Stratification of the patients according to their SARS-CoV-2 RNA and antigen status (cut-off of 200 TCID_50_/mL) in nasopharyngeal swabs.

	PCR-Negative *n* (%)	PCR-Positive *n* (%)	Total *n* (%)
Antigen-negative	72 (39.6%)	33 (18.1%)	105 (58.2%)
Antigen-positive	0 (0%)	77 (42.3%)	77 (42.3%)
Total	72 (39.6%)	110 (60.4%)	182 (100%)

**Table 2:** Sensitivity, specificity, PPV, and NPV for four different SARS-CoV-2 antigen level cut-offs for SARS-CoV-2 detection in nasopharyngeal swabs.

Cut-Off (TCID_50_/mL)	AUC	Sensitivity	Specificity	PPV	NPV
22.79	0.862 (0.8–0.923)	92% (85–96%)	81% (69–89%)	0.88 (0.8–0.93)	0.87 (0.76–0.93)
23.56	0.871 (0.812–0.93)	91% (84–95%)	83% (72–91%)	0.89 (0.82–0.94)	0.86 (0.75–0.93)
57.68	0.889 (0.839–0.938)	79% (70–86%)	99% (91–100%)	0.99 (0.93–1)	0.76 (0.65–0.84)
100	0.877 (0.826–0.928)	75% (66–83%)	100% (94–100%)	1 (0.94–1)	0.73 (0.63–0.81)
200	0.85 (0.794–0.906)	70% (60–78%)	100% (94–100%)	1 (0.94–1)	0.69 (0.59–0.77)

The numbers in brackets represent the 95% confidence interval. TCID_50_/mL: Tissue Culture Infectious Dose, AUC: area under the curve. PPV: positive predictive value, NPV: negative predictive value.

## Data Availability

The data presented in this study can become available in part on request from the corresponding author. The data are not publicly available due to data protection issues.

## Supplementary Materials

The following are available online at https://www.mdpi.com/article/10.3390/pathogens10060658/s1, Raw values for Table 2.
