# Supplementary material for: Performance of the LIAISON® SARS-CoV-2 Antigen Assay vs. SARS-CoV-2-RT-PCR
Source: Pathogens. 2021 May 26;10(6):658. doi: 10.3390/pathogens10060658 (PMC8228578; doi:10.3390/pathogens10060658)
Supplement: Supplementary file 1 [file pathogens-10-00658-s001.zip › pathogens-1203466-supplementary.pdf]

Supplement:

Raw values for Table 2

Cut-off 22.79 TCID$_{50}$/ml

Count

		RT-PCR		
		negative	positive	Total
**Antigen assay Cut-off 22.79 TCID$_{50}$/ml**	negative	58	9	67
	positive	14	101	115
Total		72	110	182

Cut-off 23.56 TCID$_{50}$/ml

Count

		RT-PCR		
		negative	positive	Total
**Antigen assay Cut-off 23.56 TCID$_{50}$/ml**	negative	60	10	70
	positive	12	100	112
Total		72	110	182

Cut-off 57.68 TCID$_{50}$/ml

Count

		RT-PCR		Total
		negative	positive	
**Antigen assay Cut-off 57.68 TCID$_{50}$/ml**	negative	71	23	94
	positive	1	87	88
Total		72	110	182

Cut-off 100 TCID$_{50}$/ml

Count

		RT-PCR		Total
		negative	positive	
**Antigen assay Cut-off 100 TCID$_{50}$/ml**	negative	72	27	99
	positive	0	83	83
Total		72	110	182
